# On the Influence of Climate on Man

**Published:** 1838-10-01

**Authors:** 


### De l'Influence des Climats sur l'Homme. Par *P. Foissac*, Docteur en Medecine de la Faculté de Paris. A Paris, 1837.

### On the Influence of Climate on Man. By *P. Foissac*, M.D.

Et plaga cœli non solum ad robur corporum, sed etiam animorum facit.

Climate contributes to the strength not only of the body, but also to that of the mind.

The author of the book of which we are now about to present a condensed analysis to our readers, originally intended it to form only a part of a much more extended work, entitled, *Histoire naturelle et Philosophique de l'Homme.* But discovering, as he proceeded, the great importance connected with such a subject as the influence of climate on man, on the production of those diseases to which he is obnoxious, as well as on the development of his intelligence, he saw that so interesting and extensive a question could not be discussed adequately or satisfactorily as a mere episode, and so determined to devote a separate volume to it.

In ordinary parlance *climate* signifies merely the temperature of a region. Medical and philosophical writers in general however give a much less restricted signification to this term. Hippocrates in his treatise *de aere, aquis, et locis,* attributes the effects of which he intends to give an account not merely to the temperature of the air, but to all its other combined qualities; not only to the degree of latitude of the soil, but to its nature, to that of its productions, and to that of the waters with which it is irrigated. Climate embraces generally the aggregate of all the physical circumstances connected with each country; in itself it constitutes this aggregate, and all the characteristic features by which different countries are distinguished, enter into the idea which we should form of climate.

"To observe," says Professor Rostan, "the simultaneous effects of light, heat, electricity, of the winds, &c. on the organic productions of the different zones of the earth, to explore the nature of this earth, to deduce from this knowledge the influence which they exercise on the physical and moral state of man, such is the wide field which climates present to our investigation." Our author defines the signification of the term climate "the aggregate of all the external, physical, and natural circumstances attached to each locality in their relation to organized beings." For convenience he intends to adopt the division of climates according to the temperature peculiar to each country into three great classes, into *warm* (chauds), *cold,* and *temperate climates.* This distinction he prefers to that of Polybius, which has been adopted by some modern writers, into warm and dry climates, warm and moist, cold and dry, cold and moist. Whatever classification of climates be adopted, it will inevitably be incomplete and defective, in as much as certain points separated arbitrarily will become confounded together by imperceptible shades.

Did the phenomenon of heat depend solely on the position of the sun with respect to the countries which it illumines, the division would be absolute, and capable of mathematical precision. But a multitude of circumstances, some known others unknown, cause a variation in the degrees of temperature, and modify the power of the solar rays in countries which are very near each other. The most simple observation shows us that at certain heights eternal snows prevail, and that in general the heat increases according as the country becomes less elevated. The limit of ice and snows upon mountains varies in proportion to the latitude; under the Equator they are perpetual only at 4,800 metres above the level of the sea, whilst at 65° they become so at 1,500 metres. The way in which a locality is exposed also produces great varieties in its temperature, and the direction of mountains has no less influence.

Every climate is again modified according as the air is calm, or according to the nature of the winds which blow over it. Siberia is colder than other countries of Norway and Russia which are as northern, but happen to be protected by mountains from the winds of the frozen ocean. The particular nature of the soil, its cultivation, productions, and the proper temperature of the globe, are so many modifying causes. Thus dry sands increase the heat; to this cause we must attribute in a great measure the excessive temperature of the deserts of Arabia and of the centre of Africa; whilst on the contrary, clayey and saline soils cool the atmosphere, as we observe in the environs of Astracan, and in several other countries. Cultivation has in general the effect of moderating extreme climates. Germany, which was very cold in Cæsar's time, is now one of the most temperate countries of Europe.

The proper temperature of the globe is one of the most obscure points of geology. Mr. Fourier has proved that the heat increases in the ratio of the depth of the strata of the earth, and he has determined with precision the measure of this increase. According to him the solar caloric penetrates by decreasing degrees into the bowels of the earth; it is compensated by an equal quantity of central caloric, which is lost at the Poles. The temperature of the Poles is little inferior to that of the planetary spaces, which is 40° below the freezing point; such also would be that of the earth, if the sun ceased to illuminate it by his rays. Thus the heat on the surface of the globe is regulated by the solar influence, and a change would be necessary in the astronomical relations of our planet, and of the body around which it gravitates, in order perceptibly to modify the return of the seasons, and the nature of the climates as now established.

Of all the modifying influences which have been as yet considered, there is none so powerful or so universal as that of water, whether it be considered as flowing by numberless canals into the entrails and on the surface of the earth, and becoming mixed with all the vegetable productions, or as accumulated in the immense reservoirs of the sea, and surrounding those continents which it appears destined one day to swallow up. The sun by its rarifying heat pumps up and reduces to the state of vapour the water of the different seas and rivers; the wind favours and assists this evaporation, and wafts into the atmosphere those humid emanations, which condensed by the cold of the night, again fall in copious dews, or which, when subjected to other physical laws, form rain, snow, and hail. The evaporation of water takes place entirely at the expense of the surrounding caloric; the vicinity of large rivers, and more especially of great seas, changes the temperature of the atmosphere. It is then very easy to account for the delightful climate of some isles in the middle of the Pacific Ocean, under the Torrid Zone; but it is not so easy to understand how, in the North, the sea-coasts enjoy a milder temperature than the interior of the plains. This however is ascertained from observation. Thus we see how the combined action of so many causes renders the classification of climates so difficult, if such classification is to be based solely on terrestrial geography. After laying down his division of climates into *hot, cold,* and *temperate,* he proceeds to the *first part,* which treats of the *Influence of Climate on Physical Organization.*

Chap I. *Of Animal Heat.* After considering the various sources of caloric in general, he comes to the subject of animal heat. Man, he says, has a temperature peculiar to him, and which continues the same at all times, and under all conditions of life. It is inherent in his organization, as the blood, lymph, and the different parts of the system. Some physiologists derive animal heat from a vital property or function as essential to man as sensibility and contractility, and designate it sometimes by the name of caloricity, sometimes by that of calorification. Others see in the faculty possessed by animals, of keeping up an almost invariable degree of temperature, merely the result of essential functions, the product of a concurrence of organs. The proper heat of the body is about from 98° to 100° Fahr. in the great cavities and in all the vital centres; this is the temperature of the liquids which circulate in the vessels, and which are thrown out by the excretory ducts. Titsing has stated (*Description de Curaçao*) that the animal heat is two or three degrees less in the people within the Tropics, whilst Davy has found it one or two degrees higher in the island of Ceylon. These contradictory facts are not borne out by the majority of observers. Gmelin, Ross, and Parry, advanced as far as 74° North latitude; there the alcohol thermometer marked 60° below zero, without this intense cold producing any appreciable diminution in the temperature of the body. Neither does external heat appear to have a more marked effect than cold on the phenomenon now before us. The Russians, Poles, Swedes, can endure the air of stoves heated to 167° F. without inconvenience. Several other experiments of the same kind satisfactorily prove that man and animals can preserve their natural temperature under very high degrees of external heat. The effects, however astonishing they may appear, are not incapable of explanation. In fact, the animal heat inherent in the human body at the time of birth, is kept up by the combined effect of several organic acts, and particularly those of respiration and circulation. Repeated experiments have shown that nine-tenths of the animal heat may be explained accurately by the quantity of oxygen which disappears, and by the formation of water and carbonic acid in the lung. The red-blooded and warm-blooded animals are those whose temperature is highest. The capacity of the thoracic cavity and of the pulmonary organs is greater in them than in any of the other classes of animals. Birds whose cellular lungs are continued into the abdomen, and into the principal parts of their skeleton, develop more heat than man and mammiferous animals. The latter again enjoy a temperature much superior to that of reptiles and fishes, whose heart has but one ventricle, whose lungs are very imperfect, and which can bear the privation of atmospheric air for a longer time. The more frequent the respiration and the more rapid the circulation is, the more heat is there developed. We may now consider what takes place in the different climates with respect to the activity of these functions.

In cold countries, and during Winter, the respiration instead of being accelerated, as some authors have stated, is perceptibly retarded; but a greater quantity of oxygen is absorbed, a more electrical and less rarified air enters the lungs. The result of this is a more considerable disengagement of caloric. A portion of this caloric is given off with the respired air; the other portion is carried with the arterial blood into the torrent of the circulation in order to be distributed to the several organs. In cold countries the necessity of great exercise and of repeated movements is felt; the physical action of the friction of the parts one against the other causes the development of caloric. Besides, exercise accelerates the circulation and respiration; the repeated aspirations of air renew more frequently the phenomenon of hematosis; these become so many new causes of an increase of animal heat.

Digestion also serves, though in a lower degree, to keep up the equality of temperature observed in the human body. Tonic and stimulating substances, all those which contain rich animalizing principles, develop a large amount of caloric. The same may be said of the use of spirituous drinks taken in moderation. It is observed that people residing in northern latitudes are great eaters, that succulent food agrees better with them, whilst those residing in southern latitudes have more appetite for vegetable diet, for sweet acidulated fruits which require less digestive power, and develop but a slight degree of caloric. Nutrition is the end and object of all the functions, and its perfect accomplishment proves their integrity. The assimilation of the chylous fluid, which circulates with the arterial blood, disengages in passing from the liquid to the solid state a quantity of heat which is constantly acting, and which penetrates the interstices of all the organs. Our author having thus far considered the subject of animal heat, next proceeds to enumerate the different kinds of food used by the different nations of the known world. These, as being matters with which our readers are already sufficiently well acquainted, we shall pass over, and proceed to the functions of respiration and circulation. He here observes that, notwithstanding the constant uniformity of atmospheric air under all latitudes, with respect to chemical composition, its physical qualities are extremely variable. Cold renders the air more dense: the introduction of a greater quantity of this fluid into the lungs stimulates the functions of these organs, and also increases their capacity. By a contrary action the chest of southern nations attains less expansion. He here notices the greater quickness of pulse in persons residing between the Tropics, as stated by some travellers—this increased frequency of pulse however in intertropical climates has not been observed by modern observers. On the function of *absorption*, which forms the subject of the 4th chapter of this work, he remarks that it becomes more active according as the system stands more in need of reparation; women and children absorb with more activity and energy than adult and old men: atmospheric pressure, caloric, electricity, sleep and rest, also seem to encrease the energy of this function. An empty state of the vessels, hunger, thirst, fatigue, and debility, act in the same way; parts denuded of the cuticle, and those parts where the cuticle is very thin, also possess increased absorbing powers. In this way, says our author, we may more readily explain how a person enfeebled by debauchery, fatigue and loss of sleep, is more liable to catch the venereal disease; and how individuals, exhausted by the same causes and by the debilitating effects of fear, are more exposed to the action of contagion. Hence in those countries where those destructive epidemics prevail, the necessity of preserving calmness and serenity of mind, and of avoiding all those excesses which may tend in any way to dissipate the strength. Our author next considers the secretions; those two only, however, which are influenced by climate, viz., the cutaneous transpiration and the urine. After detailing the experiments of Sanctorius on the cutaneous exhalation, with which and with the results of which our readers are already acquainted, he remarks that the cutaneous transpiration undergoes from the effect of temperature more considerable variations than the pulmonary transpiration, it being even probable that the one is destined to replace the other. This close correspondence will account for the frequency of pulmonary diseases occasioned by the impressions of cold made on the surface of the skin. There is one case however where the cutaneous and pulmonary transpiration are simultaneously very profuse, namely, after violent bodily exertion, which accelerates the respiration and circulation at one and the same time. According to Sanctorius the insensible transpiration is more copious at night than by day; he estimates the quantity of serum which a healthy man loses by night in seven hours' sleep, at fifty ounces. The experiments of Keil and Gorter go to prove however, that this loss does not exceed sixteen ounces, and that in Holland and England it is greater by day than by night. All observers agree in stating that a profound and tranquil sleep facilitates this function, whilst it is interrupted by a restless and sleepless night. The cutaneous exhalation diminishes when the stomach is empty, or when it is too full; substances easy of digestion render the respiration easy; generous wine used moderately favours the functions of the stomach and skin. Our author mentions that he has known a great number of persons, in general in the class of hypochondriacal and hysterical subjects, in whom all the excretions were scanty, whose skin, constantly dry and parched, appeared incapable of perspiring. In these persons, who by the way were usually valetudinarians, the pulmonary transpiration was extremely active. The breath of such persons contracts a peculiar odour, which, though not always fetid, imparts deleterious qualities to the air of the apartments, and renders their habitations insalubrious.

Our author having considered in the *first part* of his work the organic modifications which man undergoes under the influence of different climates, now comes to consider the effects of this influence on health. This part he prefaces by some general considerations. He first considers the opinion of the ancient philosophers with respect to the origin of diseases. They would have it that diseases were in a great measure the result of luxury and of those artificial wants created in the state of society. Though there certainly is some truth in this, yet to a close observer it must appear manifest that diseases are the inevitable consequence of our organization. Physical sensibility is the source, the result, and the condition of life. This great function is constantly supported by external agents, and becomes extinct, when deprived of them. Without air, heat, and aliment, the organs become changed, sensibility is destroyed, and the individual dies. If this privation is not total, if the natural composition of those agents is only altered or modified, life may not be destroyed, and may still be dragged on in a languid state. The faculty of perceiving agreeable or painful impressions is one and the same, and when beings sensible to pleasure were created, these same beings were also subject to suffering.

As a further proof that diseases are not solely attributable to the refinements and luxuries of civilized society, he adduces instances of several tribes of savage nations, as also of some species of the lower animals, among which diseases, and chiefly diseases of an epidemic character, prevail. In the sultry climates of Asia and Africa, where the absence of social institutions retains man in a state of eternal infancy, the mortality is more terrific than even in the midst of civilized Europe. It is evident however that there are less connate infirmities among savage nations. But whilst it is admitted that original mal-formations which depend almost all on a scrofulous taint, are more frequent in civilized countries, it should be recollected that in savage nations every malformed child is quickly destroyed and thus prevented from forming a part of society. If the progress of civilization has extended the field of disease by affording an unrestrained course to a thousand inordinate passions, we should recollect on the other hand, that we are indebted for the greatest blessings to the advancement of science, and more especially that of medicine. The former ravages of epidemics among us are now considerably mitigated. The application of the precepts of hygiene, by drying up the sources of infection, has rendered certain diseases less frequent and less fatal.

With respect to the etiology of diseases, they were attributed in the infancy of society to the anger of the gods; superstitious man endeavoured to appease them by sacrifices and expiations. Thus the priests who were regarded as the depositaries of the divine power, were also the first physicians. Though in the hands of such practitioners nothing but expiatory offerings and magic forms of words met the vulgar eye in the treatment of disease, still those medicinal and hygienic resources, either suggested by the instinct of the patients, or sanctioned by the authority of experience, were by no means neglected. Thus we see that among the Hebrews faith alone, or mere words, or the imposition of hands, did not suffice for the cure of leprosy; Moses was careful also to prescribe the most judicious regimen and that which best suited the nature of the climate. Elyseus cured the Syrian general Naaman by making him bathe in the waters of the Jordan. The soothsayer Melampus cured Iphiclus of impotence by the oxide of iron. Sobriety, continence, and gymnastic exercises, which comprised the therapeutics of Pythagoras, were more effectual in the treatment of disease than the sacrifices and other superstitious observances. Hippocrates, alluding to a disease somewhat prevalent among the Scythians and which they regarded as a punishment from the Deity, observes, that this disease was just as divine as others; all diseases have a natural cause; this cause, no doubt, oftentimes escapes us, and even though by close observation we may succeed in discovering it, we cannot always discover the connexion between the cause and the effect.

Etiology is confessedly one of the most useful and instructive studies in which a physician can engage, as it is on it the most solid foundations of therapeutics rest, as also the most positive and most important notions of hygiene. One of the best attested and most general influences is that exercised by climate on the production of diseases and on their different phases.

We now come to that part of his work where the author considers the *diseases of cold climates.* The most general effect of cold is to diminish the size of bodies by approximating their molecules. When applied to the animal economy it contracts the skin, drives towards the centre those fluids which the organ of circulation projected to the periphery. The cutaneous exhalation becomes less copious, rather in consequence of the density of the air, than of its low temperature. The pulmonary transpiration replaces it, and compensates the want of activity of the cutaneous functions. The atmosphere of northern regions is more dense, and more electrical; a greater quantity of air is introduced under the same volume into the respiratory organ, and so yields more oxygen and more heat to the blood and to the entire system. When the cold is too intense, the vital reaction being insufficient, the lungs absorb but a very small quantity of oxygen for the support of life. When this occurs, the venous blood, no longer converted into arterial blood by the contact of the air, penetrates the brain, ceases to stimulate it, the interruption of the functions of the sensitive centre causes the action of the heart and the function of respiration to cease, and life becomes extinct. One of the most constant effects of cold is a treacherous sleep, the fore-runner of death. The cold more particularly affects those organs placed at the periphery of the body, and which are more removed from the centre of circulation. By constringing the ultimate vascular ramifications it drives the blood chiefly to the head. Most of the persons who fall victims to intense cold die apoplectic, the vessels of the brain being usually found gorged with venous blood. In the same way we may account for the long-continued sleep of the inhabitants of nothern countries, and the comatose and lethargic affections so frequent among them, and the violent headaches to which we are told by Linnæus the Laplanders are subject. It is unnecessary here to dwell on the chapped skin, those affections of the hands and feet, called chilblains, which are not unfrequent among us, even during our comparatively mild Winters, but are more general and more severe in northern countries; very frequent instances occur amongst them of gangrene occasioned by cold, the nose, ears, fingers, toes, feet, hands, and sometimes entire limbs becoming sphacelated. It has been already stated that the cutaneous transpiration is considerably diminished during Winter, and that the pulmonary transpiration replaces it in northern climates. Hence, from the well-known physiological law, that the more excited an organ is, the more liable is it to disease, we shall not be surprised to find a great number of cases of angina, bronchitis, peripneumony, and pleurisy. The intestinal mucous membrane, which is less frequently excited, is seldom attacked with inflammation. We cannot help saying that we were not a little amused with a remark of our author's here, which is, that "it sometimes happens that the pulmonary exhalation scarcely arrives in the air-passages, when it is converted into a multitude of small icicles which irritate and lacerate the pulmonary mucous membrane." This is a morceau of pathology, which we really cannot swallow.

The eyes are no less exposed than the lungs to the terrible influence of cold. The snow which envelops the country on every side for several months in Russia, Norway, and Poland, the sharp winds from the frozen ocean which strike directly on the palpebral mucous membrane, the dense smoke which prevails in the dens of the Laplanders and Esquimaux; the sand from the deserts of Siberia which is wafted to and fro' in the air, occasions ophthalmias, which, in the north of Asia, of Europe, and of America, attack not only individuals, but whole nations. Thus all the Laplanders are remarked for having red, swollen and ulcerated eye-lids; they can scarcely endure the light of day, and generally walk with one hand placed at the lower part of the forehead, so as to avoid the impression of the solar rays. Amaurosis and cataract are very common in Russia, Poland, and all northern countries.

When we consider that the sanguineous temperament, an irritable constitution, the abuse of alcoholic liquors, and the prolonged application of cold and moisture combined, are the causes most calculated to produce rheumatic affections, we shall not be surprised at finding that these diseases do prevail very generally in almost all northern countries.

Notwithstanding the filthy habits of the Polar nations, who, at the approach of their long Winters, bury themselves in their subterraneous habitations with their families, their dogs, and their reindeer; notwithstanding also the bad quality of their food and drink, and the oil and fats with which they rub their bodies, cutaneous diseases are not so numerous among them as in warm climates, which is to be attributed no doubt to the little excitement and vitality of the skin. It is an observation worthy of being remembered, that when the diseases of equatorial regions spread into cold countries, they sometimes make great havoc there, and are much more difficult of cure than in warm climates. Small-pox sometimes assumes an epidemic form in those cold countries, and then proves a terrible scourge. In fact it is to its malignity that the depopulation of Greenland and of Kamschatka has been in a great measure attributed. Epilepsy and hypochondriasis, according to some travellers, are not uncommon among the inhabitants of the Polar regions; thus, whilst the Laplanders have their feelings blunted and well-nigh obliterated, whilst the savages of North America, insensible to physical pain, mutilate themselves and shed their blood amidst shouts of boisterous joy, these same individuals, timid and pusillanimous, fall, it is said, into violent convulsive fits on the slightest impression made on them, at the least unexpected noise. The women of Kamschatka, Lapland, and Iceland, are subject during the menstrual period to hysterical and other nervous affections. Civilization has introduced into the countries of the North no less nervous diseases than into temperate climates. They are very frequent in Russia, Poland, and Sweden. Denmark is one of the European nations where suicide, that moral distemper of civilized states, reckons the largest number of victims.

The combination of cold and moisture is, of all atmospheric conditions, that which exercises the most fatal influence on the system. It is to the influence of this cause we must attribute scrofula and all those chronic diseases which, under a variety of forms, affect the lymphatic system. Scrofula has become according to our author more frequent and of a worse character since the appearance of syphilis. The latter disease has now spread very extensively over both the high and low classes in Russia, Sweden, and over the northern parts of Asia. There are few countries where one meets with more persons without noses than in Poland.

Plica, a disease which prevails endemically in Poland and in some parts of Russia, our author considers to be a particular form of scrofula, which is often preceded by symptoms of articular rheumatism; the repercussion of this disease has been frequently followed by the most disastrous consequences, as apoplexy, phthisis pulmonalis, epilepsy, caries ossium, and amaurosis. Its treatment is the same as that of scrofulous diseases in general, sudorifics, depuratives, and antiscorbutic remedies, seconded by strict attention to hygienic rules, without which no treatment is of any avail. Having now enumerated the principal diseases to which the inhabitants of the northern climates are obnoxious, he makes one remark which, as being of practical importance, we extract in our author's own words : "The lymphatic and muscular constitutions of the North are less sensible to stimulants of every kind, whether internal or external ; from this less degree of susceptibility results the necessity of employing more active remedies and larger doses of these remedies. In Russia, Spanish pepper mixed with brandy is regarded among the lower classes as a universal panacea. Hellebore and aconite also enjoy a high reputation. Opium, cicuta, belladonna, tartar emetic, and quinine, produce their effects more slowly, and less characteristically than in temperate climates." To these general observations, however, there are numerous exceptions. We shall now follow our author in his observations on the diseases of hot climates. After noticing the effects of caloric on bodies in general, he remarks that the animal economy also, though withdrawn from the dominion of physical agents by the vital principle, still receives certain modifications from their influence. Thus, under the agency of an elevated temperature, the motion of expansion from the centre to the circumference being further aided by the diminished density and consequently diminished pressure of the surrounding atmosphere, all the fluids of the body are attracted towards the skin. This organ is penetrated by more numerous as also by larger branches of the lymphatic, sanguineous and nervous structures; this increase of vitality, sensibility, and exhalation renders skin diseases more severe and more numerous.

In warm climates, the air being rarer and more expanded, the lungs absorb less of oxygen. The proportions of the venous system will thus preponderate over those of the arterial; the liver also, whose business is to remove hydrogen from the blood, will become very active. The bile will be secreted in greater abundance, and the system of the vena portæ will become more developed. The result of this excess of action and of vitality will be an increase of disease, as well in the structure as in the functions of the biliary apparatus. Nor is this stimulating influence of heat and light confined merely to the skin; they also excite the brain and nervous system. It may be observed also that though heat by relaxing the tissues disposes to repose, and keeps the senses in a state of languor and dullness, still persons sleep less in warm climates; this privation of refreshing sleep our author considers to be one of the causes which renders cerebral diseases and idiopathic nervous affections in general more numerous and more severe.

Under the Tropics only two seasons are known: the hot and the rainy season ; in the latter, which lasts about three months and in which the temperature is considerably diminished, pulmonary catarrhs, pleurisies and peripneumonies, soon followed by hepatization of the lung, are very prevalent. These inflammations are occasionally followed by pulmonary phthisis, which though by no means so common here as in the temperate climates of Europe, yet is occasionally observed in the Antilles and under the Equator, where it assumes a very acute and rapidly fatal character.

Small-pox derives its origin from the hot climates of Arabia, Abyssinia, and Ethiopia, where it is endemic. Measles has the same origin. Lepra, the most formidable of all cutaneous diseases, derived its birth from the burning regions of Arabia, Syria and Egypt.

Our author ventures an opinion here on the nature of lepra: he thinks it not improbable that it is a scrofulous degenerescence occasioned by climate; this view of the matter he conceives to be strengthened by the fact that the constitution where the lymphatic system predominates is that most obnoxious to it. Elephantiasis, which is a very rare disease in temperate climates, is endemic in hot countries. It is very common among the negroes. All parts of the body may be affected with it; it is more usually seated however in the extremities and chiefly in the lower extremities. The Indians, as a cure for this disease, cut up into small bits a species of lizard called by them anolis, and swallow three of these reptiles in the raw state every morning, till they are perfectly cured.—The next disease noticed by our author is one called *pians*, which consists of tubercles resembling the large pustules of small-pox. This is considered by some as one of the most formidable varieties of syphilis. It is not only propagated by hereditary transmission, but even the mere momentary contact of a person labouring under it is sufficient to communicate it, or even touching any object which belonged to him. Mercury, sudorifics, and arsenical preparations are the remedies found to be most successful in its treatment.

Hepatic disease from the excited state of the liver, gastro-enteritis and dysentery from repercussion of the cutaneous transpiration and the immoderate use of fruit, are very prevalent in hot climates. It is unnecessary here to follow our author in his descriptions of cholera and of the yellow fever, so prevalent in those climates. The constant excitement of the nervous system, the putrefaction of animal and vegetable substances, assisted by the burning influence of a tropical sun, very commonly occasion cerebral diseases in different forms, as also those fevers called putrid or adynamic, malignant or ataxic. Intermittent fevers, whether of a simple character or complicated with some gastric or hepatic affection, are so common in those climates that they become associated with all diseases in their different stages, and assume all characters and all types. Neuroses of every species and of the worst character, such as cramps, convulsions, tetanus, epilepsy, hysteria, hypochondriasis, catalepsy, paralysis, etc. are of very frequent occurrence in tropical climates. Exposure to the atmosphere when cooled, is particularly fatal to children, and gives rise to fatal convulsions, trismus and tetanus. Acute diseases we often see ushered in by violent convulsive paroxysms.

Dropsy, in its various forms, occurs with considerable frequency under the tropics. This disease may be occasioned by the humid state of the atmosphere, in our author's opinion, or by the vegetable and relaxing quality of the food, or what appears to us more probable, by obstructions in the liver or the abdominal viscera. Scurvy, though it exercises its ravages chiefly in cold climates, does not however entirely spare the equatorial regions. It is said to be so frequent in the Antilles that there are some constitutions there decidedly scorbutic. When concluding this part of his work our author remarks that the treatment required for these extraordinary diseases of hot climates must be prompt and energetic as the diseases themselves; that bloodletting, so useful in other places, is seldom indicated or efficacious in tropical countries, even at the onset of acute diseases, and that tonics are in general very salutary. In the most formidable diseases of the skin, he says that in those cases where the resources of art are not entirely powerless, it is from hygiene that the best curative means are to be derived. The juices of plants, wholesome diet, from which fish, eggs, and salted meat are excluded, baths, and attention to cleanliness succeed more frequently than pharmaceutical remedies. Convulsive diseases and intermittent fevers call for prompt as well as strong doses of sulphate of quinine. Camphor, sal ammoniac, tartar emetic, and large blisters are also employed with advantage. The avoidance of excess at table, as also of those enervating indulgences so much practised in hot countries, guarding against the noxious influence of exposure to the cold and humidity of the night air, as well as removal from the mischievous effects produced by the decomposition of animal and vegetable substances, are most imperatively enjoined by the author.

In chap. 7, where he treats the subject of mortality, our author makes some curious and not uninteresting remarks; and first, with respect to children dead-born he says, that the ratio of the dead-born to the births is but very imperfectly known, as it varies prodigiously, according to the different climates: thus there is one death to 19 births in Paris, one to 20 at Berlin, one to 24 at Vienna, one to 27 in London, one to 36 at Stockholm. The mean or average for all Europe is one to 22. The number of dead-born is greater in Winter than during the other seasons, in cities than in the country, and three times greater in illegitimate births. Our author here mentions a very surprising fact with respect to the number of dead-born in both sexes; it is that the mortality is greater among boys than among girls; this law has been found to hold good throughout every country in Europe, the relative numbers being 13 and 10. The mortality is found to be less, generally speaking, in temperate climates than in those where the temperature is in the extreme. England he finds to be the country where the mortality is least, there being one birth to thirty-five inhabitants, and only one death to fifty-eight.

From the researches of statistical writers, it appears that all over Europe the maximum of deaths occurs towards the close of Winter, and the minimum towards the close of Summer. With respect to the *mean duration of life*, it was in France before the revolution twenty-eight years nine months; it is calculated to be thirty-two years and a half. In England it is thirty-three years. Death carries off a great number of children of both sexes at a premature age; the tenth perishes a month after birth; a fourth is carried off after the first year; a third at the end of the second; there scarcely remains one-half at the end of twenty years. Our author very justly attributes the increase in the average duration of life to the introduction of vaccination. With respect to the *probabilities of life*, our author observes that the chances of death are not the same for both sexes at different ages. During the first, and even the second year after birth, more boys die than girls; then the number of deaths in the two sexes becomes equal till the period of puberty. Then more females die than males; the number of the latter preponderates at the age when violent passions prevail, viz. between the age of twenty and thirty. At no period of life, with the exception of the year after birth, is there such great mortality. The turn of life is fatal to some women; but this epoch once safely passed, their health becomes confirmed so as to resist the incroaches of disease for a considerable number of years. They attain a considerable old age more generally than men; yet there are not so many centenarians found among them. The remaining part of the author's work consists of some sections on the art of prolonging life; some observations on the influence of climate on the morals, &c. As there does not appear much that is novel in these chapters, we shall close our analysis here.

